# Clinical and genetic aspects of Bardet–Biedl syndrome in adults in Norway

**DOI:** 10.1186/s13023-025-03641-3

**Published:** 2025-03-14

**Authors:** Cecilie Fremstad Rustad, Ragnheidur Bragadottir, Kristian Tveten, Hilde Nordgarden, Jeanette Ullmann Miller, Pamela Marika Åsten, Gisela Vasconcelos, Mari Ann Kulseth, Øystein Lunde Holla, Hanne Gro Olsen, Charlotte von der Lippe, Solrun Sigurdardottir

**Affiliations:** 1 Centre for Rare Disorders, Oslo University Hospital, Oslo, Norway; 2 The Medical Faculty, University of Oslo, Oslo, Norway; 3 Department of Ophthalmology, Oslo University Hospital, Oslo, Norway; 4 Department of Medical Genetics, Telemark Hospital Trust, Skien, Norway; 5 National Resource Centre for Oral Health in Rare Disorders, Lovisenberg Diaconal Hospital, Oslo, Norway; 6 Department of Medical Genetics, Oslo University Hospital, Oslo, Norway

**Keywords:** Bardet–Biedl syndrome, Adults, Genotype, Phenotype, Deep intronic variant, Kidney disease, Obesity, Oral anomalies, Retinitis pigmentosa

## Abstract

**Background:**

Bardet–Biedl syndrome (BBS) is a rare nonmotile ciliopathy characterized by retinal dystrophy, polydactyly, obesity, genital anomalies, renal dysfunction, and learning difficulties. The objectives were to describe the retinal, oral, and metabolic characteristics relevant to adults with BBS as well as the prevalence of genetic variants.

**Methods:**

A cross-sectional study of 30 adults with BBS (15 males, 15 females, mean age 39.8 ± 13.6 years) was recruited from a single centre for rare disorders in Norway. Participants attended a one day hospital visit including medical (blood pressure, body mass index), ophthalmological and oral examinations. Blood samples were collected and genetic analyses were performed.

**Results:**

Age at diagnosis varied from one year to 30 years. The incidence of overweight/obesity, hypertension, kidney disease, and diabetes mellitus was 82%, 67%, 27%, and 23%, respectively. All had retinitis pigmentosa. Prior to the study, 14 participants (47%) had confirmed extinguished electroretinography. Eleven participants were examined with electroretinography during the study period, and all had extinguished electroretinography. 50% perceived light, 23% saw hand motion, and one participant did not perceive light. Oral anomalies were identified in 77% of the participants, including abnormal palates (58%), crowded teeth (50%), and small teeth (60%). A genetic cause was identified in all participants, most commonly in *BBS1* (*n* = 11) and *BBS10* (*n* = 9). Other variants were found in *BBS5*, *BBS7*, *BBS9*, and *MKKS.* In addition to exon-located variants, a novel deep intronic variant causing mis-splicing was identified in *BBS7*.

**Conclusions:**

A multidisciplinary examination is important for proper management of BBS. The genotype and phenotype of this sample were heterogeneous, including kidney failure, genital anomalies and obesity. Genome sequencing increased the likelihood of identifying the genetic cause. In BBS populations, the patients will benefit from testing or reanalysis, preferably with genome sequencing, including searching for deep intronic variants.

**Supplementary Information:**

The online version contains supplementary material available at 10.1186/s13023-025-03641-3.

## Introduction

Bardet-Biedl syndrome (BBS) (OMIM: PS209900) is a nonmotile ciliopathy disorder caused by pathogenic variants in at least 27 different genes [1–5]. High genetic heterogeneity gives rise to a wide range of clinical manifestations (i.e., signs and symptoms) that affect different body systems. The clinical diagnosis of BBS requires the presence of four major features or three major and two minor features [6]. Rod-cone dystrophy (eye), postaxial polydactyly, obesity (truncal), genital anomalies (hypogonadism), renal anomalies (kidney), and learning difficulties have been described as major features, whereas diabetes mellitus, ataxia, hypertonia, oral and dental anomalies, cardiovascular anomalies, liver disease, facial dysmorphism, syndactyly and olfactory dysfunction have been described as minor features [4, 7, 8]. Genetic testing may be crucial for confirming or refuting a diagnosis of suspected BBS [9]. With the increasing availability of genetic testing, genetic confirmation of BBS has become possible at an earlier age. Recent research has shown that the median age at diagnosis is approximately six years [7]. BBS is considered a rare disorder, affecting ∼ 1/13,500 to ∼ 1/160,000 individuals worldwide [8, 10–12]. Isolated populations have a greater incidence [12].

BBS represents a challenge to the health care system due to its heterogeneity in the clinical presentation with for instance retinal, renal, and endocrinological diseases and long-term treatment needs [13]. Individuals with BBS may suffer from chronic kidney disease [14, 15], which occurs in 31% of children and 42% of adults [16]. The majority of individuals with BBS have oral/dental anomalies due to abnormal embryonic orofacial and tooth development [17].

Currently, there is no cure for BBS, and treatment is only symptomatic. Management and surveillance focus on addressing the major features of the syndrome [5]. The most common feature (although not present in all individuals) is rod-cone dystrophy, which causes night blindness, loss of peripheral and central vision, and eventually blindness [18, 19].

Several studies concerning the management of BBS, including weight management [7], kidney disease [14, 20], and dental management [17], have been published. The use of setmelanotide for obesity and weight management in BBS has been approved by the Food and Drug Administration (FDA) and the European Medicines Agency (EMA) [21, 22]. Obesity is common in BBS patients, often beginning in early childhood and persisting throughout adulthood. There are several possible mechanisms for obesity in BBS, including abnormal cilia, insulin resistance, leptin dysregulation, and the leptin–melanocortin receptor pathway, affecting both central appetite control and energy expenditure [11, 23, 24].

BBS genes encode proteins of the cell cilium and basal body [25], and BBS proteins are important for intraflagellar transport. Pathogenic variants in *BBS1*, and *BBS10* account for 45% of all BBS cases according to a meta-analysis of BBS in 899 individuals [1]. Similarly, a recent study by Nasser and colleagues [25] revealed that *BBS1* and *BBS10* were the genes most commonly associated with BBS. Increasing knowledge about the genetic variations and phenotypes of BBS can have significant implications for the clinical management of BBS. The meta-analysis which included 85 articles demonstrated a genotype-phenotype relationship in BBS [1]. Individuals with BBS caused by variants in *BBS2*,* BBS7*, or *BBS9* had a greater incidence (> 60% of cases) of renal anomalies than those with BBS caused by variants in *BBS1*,* BBS4*, or *BBS8* [1]. In a study of 350 individuals with BBS in the United Kingdom, approximately 50% had a *BBS1*-related disorder, which was associated with less severe kidney disease than, for example, *BBS10*-related BBS [16]. A retrospective study of 67 individuals with BBS caused by variants in *BBS1* or *BBS10*, recruited from six countries, revealed that the onset and progression of retinal degeneration occur earlier and faster in *BBS10*-related BBS than in *BBS1-*related BBS [26].

There is a paucity of clinical studies on BBS that focus on multidisciplinary approaches [5]. Multidisciplinary health care is necessary for the timely management of clinical manifestations of BBS, including diseases of the eyes, kidneys, cardiovascular system, endocrine organs, and liver. Dental anomalies are also common characteristics of BBS. Individuals with BBS in Norway do not have a national, structured multidisciplinary follow-up. The objectives of this study were to describe the retinal, oral, and metabolic characteristics as well as the prevalence of genetic variants in 30 adults with BBS in Norway, with the aim of improving disease management.

## Methods

### Study design

This was a national cross-sectional study carried out at Oslo University Hospital, Oslo, Norway, and the National Resource Centre for Oral Health in Rare Disorders, Lovisenberg Diaconal Hospital, Oslo, from January 2022 to March 2023. Clinical evaluations were scheduled three months in advance to coordinate activities and travel arrangements. The clinical assessment took approximately five hours (ocular, oral, physical examinations, and self-reports). Participants received a written note after their clinical evaluation and a telephone consultation when blood tests and genetic results were available.

### Recruitment

Participants were recruited through the Centre for Rare Disorders, Oslo University Hospital in Norway, and through advertisements on the Norwegian Bardet–Biedl Syndrome Organization’s webpage. Participants were 16 years or older. Forty-six individuals with a registered clinical and/or genetic BBS diagnosis at the Centre for Rare Disorders were invited to participate in this study. During the study period, one individual was registered as deceased shortly after the invitation letters were distributed. Thirty-one individuals agreed to participate and were included after providing written informed consent. One individual was excluded due to not fulfilling the clinical criteria for BBS, and upon genetic evaluation, another genetic cause was identified for the person’s condition. Thus, the response rate was 68% (30/44). Comparisons between the participants with BBS (*n* = 30) and nonparticipants (*n* = 14) revealed no differences regarding age (*p* = 0.660) or sex (*p* = 0.88).

### Demographics and medical history

Detailed demographic information was obtained, including sex, age, marital status, education, number of children, and family history of BBS (i.e., first-degree relative with a BBS diagnosis). Information was obtained on medical history through self-reports, including (but not limited to) illnesses or diagnoses related to the kidneys, liver, neurological system, and heart; cancer; retinitis pigmentosa (RP); diabetes mellitus; any surgeries in the past; current medications; and any psychological problems over the last year (e.g., depression, anxiety, or other psychological illness).

Clinical examinations were performed by one researcher (CFR) for 73% of the sample. This examination included height and weight measurements by using a Seca 704 s (Seca GmbH & co. KG., Hamburg, Germany). Due to the delayed delivery of the Seca 704 s, the weight of some participants was measured using a digital scale with a precision of 0.1 kg, and their height was measured using a measuring tape against a wall. Participants were wearing light clothing during weight measurements. Four participants had home visits, and their height and weight were recorded with locally available instruments.

Body mass index (BMI) was calculated from the participants’ height and body weight (BMI = kg/m^2^). BMI values were classified into three groups: normal weight (BMI > 18.5 kg/m^2^, < 25 kg/m^2^), overweight (BMI ≥ 25 kg/m^2^ but < 30 kg/m^2^) and obese (BMI ≥ 30) [27]. Central obesity was evaluated by looking at the protrusion of the abdomen, waist and hip measurements were not taken.

Blood pressure was measured with an A&D UA-611 upper-arm blood pressure monitor (Tokyo, Japan) with either an adult cuff or a large cuff (arm sizes from 22 cm up to 45 cm). Blood pressure was measured while the participants were in a seated position. Hypertension was defined as systolic blood pressure (SBP) above 140 mmHG and/or diastolic blood pressure (DBP) above 90 mmHG, according to the European guidelines [28], in addition to those who were already on antihypertensive treatment.

### Blood samples

We collected blood samples for genetic analysis and analyses of full blood count, renal, liver, and thyroid profiles in addition to total cholesterol, low-density lipoprotein (LDL), and triglyceride levels. The participants were informed that blood samples should be taken after an overnight fasting period. The blood samples were collected at their general practitioners’ office or at a hospital chosen by the participants and then sent to the closest laboratory for analyses of the ordinary blood tests.

### Ophthalmological examination

Ophthalmological examination, including best-corrected visual acuity (Clear chart), intraocular pressure, slit-lamp anterior examination, biomicroscopy, and wide-field fundus photography with fundus autofluorescence (Optos), was performed for 26 participants. Electroretinography (ERG) was performed if not previously recorded. Ophthalmological history, including cataract and cataract surgery, was collected. All eyes were dilated with cyclopentolat 1% and metaoxedrin 10%. All eye examinations were performed by one ophthalmologist (RB).

### Oral examinations

Data regarding oral manifestations were collected at the National Resource Centre for Oral Health in Rare Diagnoses, Oslo, Norway. Both a dentist (HN) and a speech-language pathologist (PÅ) performed the oral examinations. Panoramic X-ray and clinical photographs were taken of all participants and independently evaluated by a specialist in orthodontics (GV) after the examination.

The facial profile (straight, convex, concave) and mandibular angle were measured as described by Proffit and Ackerman [29]. The mandibular angle is increased when it is more than 30 degrees and decreased when it is less than 25 degrees [29].

In the evaluation of palatal morphology, the palate was defined as high arched when the height of the palate at the level of the first permanent molar was more than twice the height of the teeth [30], and the palatal width was evaluated on photographs and defined as narrow based on the distance between the first permanent molars [30].

The optimal overjet range for average dentition is considered to be 2–4 mm, and the ideal overbite range is considered to be 2–3 mm [29]. Excessive vertical overbite was registered when it was ≥ 4 mm, and overjet was registered as normal (2–4 mm), increased when > 4 mm, and decreased when < 2 mm according to clinical practice. Additionally, an open bite (lack of tooth contact) and crossbite (misalignment of one or more teeth in any part of the mouth, resulting in the upper teeth fitting inside the lower teeth) are well-established clinical terms.

Crowding of teeth was defined as mild (< 2 mm of dental arch length discrepancy), moderate (2–4 mm of dental arch length discrepancy), or severe (> 4 mm of dental arch length discrepancy) [29]. Individuals who had a high palate and/or crowded and/or small teeth were considered to fulfil the oral/dental minor criteria.

### BBS genotyping

The results from any previous genetic analyses were collected. A new genetic analysis was performed for all participants except for one with a recent test result and for one who did not provide a blood sample. Participants, or in a few cases their legal guardians, were offered genetic counselling. Whole exome sequencing was performed using Twist Exome 2.0 (Twist Bioscience) and NextSeq500 (Illumina). Twenty-seven genes associated with BBS and related conditions were analysed. Whole-genome sequencing was performed using a TruSeq DNA PCR Free Kit (Illumina) and a NovaSeq 6000 (Illumina). Blood samples for RNA analysis were collected in PAXgene tubes, and mRNA analysis was performed using RT‒PCR followed by cDNA sequencing.

One participant had a confirmed recent test result, for that analysis exome sequencing had been conducted (Agilent SureSelect Human All Exon) with a gene panel including genes related to retinal dystrophies. We analysed a skin biopsy from a parent of one participant to evaluate the splicing effect of a variant.

### Statistical analyses

Descriptive statistics are presented as the means (M) and standard deviations (SD) for normally distributed data analysed by the Shapiro‒Wilk test for normality. The medians with interquartile ranges (IQRs) are presented for data that were not normally distributed. Categorical data are expressed as percentages. The statistical analyses were performed with SPSS version 29.0.0.0 (IBM Corporation, Chicago, U.S.). Graphical illustrations were created with GraphPad Prism (v.10.1.0 Graph Pad Software, Boston, MA, USA).

## Results

Thirty participants from 27 families were included in the study. The demographic and clinical characteristics of the total sample (*n* = 30) are summarized in Table 1. The sample consisted of 15 males and 15 females with a mean age of 39.8 years (SD = 13.6). Most participants had been clinically diagnosed with BBS in childhood at a median age of nine years (IQR 13.5). The majority had completed high school (77%), and three people (10%) had a university education.

**Table 1:** Demographics of the study sample (*n* = 30)

	M (SD)	Range
Age at inclusion in years	39.8 (13.6)	20–69
Age diagnosed with BBS (Median, IQR)	9.0 (13.5)	1–30
Height (cm)	172.0 (10.5)	154–194
Weight (kg) (Median, IQR)	104.3 (41.4)	72–247
Body mass index (Median, IQR)	34.4 (11.9)	24.9–71.8
	**n (%)**	
Sex (males)	15 (50)	
Single	26 (87)	
High school education	23 (77)	
Currently employed	5 (17)	
Disability benefits	25 (83)	
Family members with BBS	10 (33)	
Being a parent to a biological child	1 (3)	
Taking daily medication	25 (83)	

Abbreviations: BBS = Bardet-Biedl syndrome

**Fig. 1:**
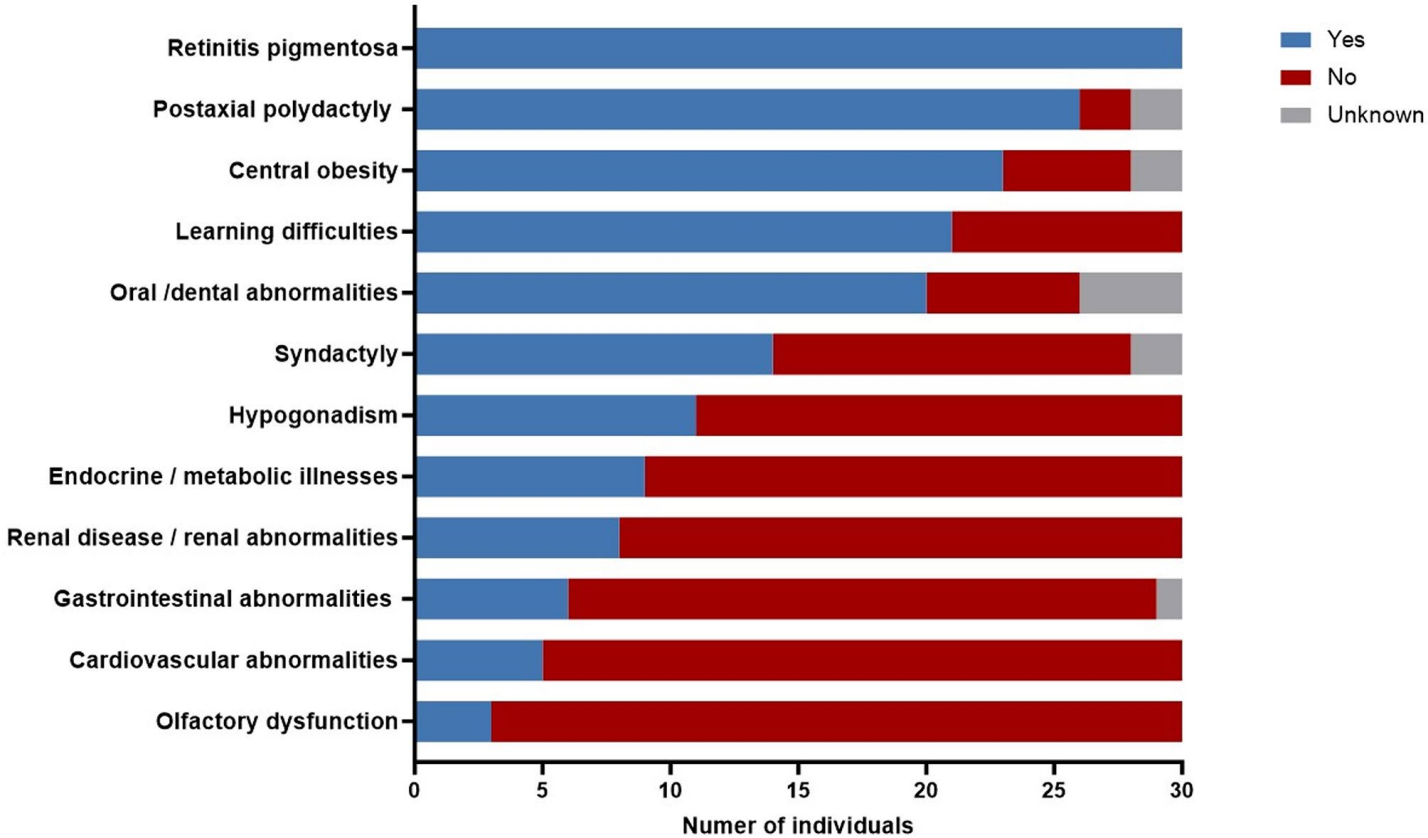
Frequency of the major and minor features of Bardet-Biedl syndrome

Assessments of the clinical diagnosis of BBS (major/minor features) were conducted, and 28 participants fulfilled the criteria, i.e., either four major features (22/30 participants) or three major and two minor features (6/30 participants). In two individuals, we could not confirm or exclude whether they were born with postaxial polydactyly or not, and could therefore not conclude if they fulfilled the criteria. The details of the major and minor features observed in the participants are illustrated in Fig. 1.

**Fig. 2:**
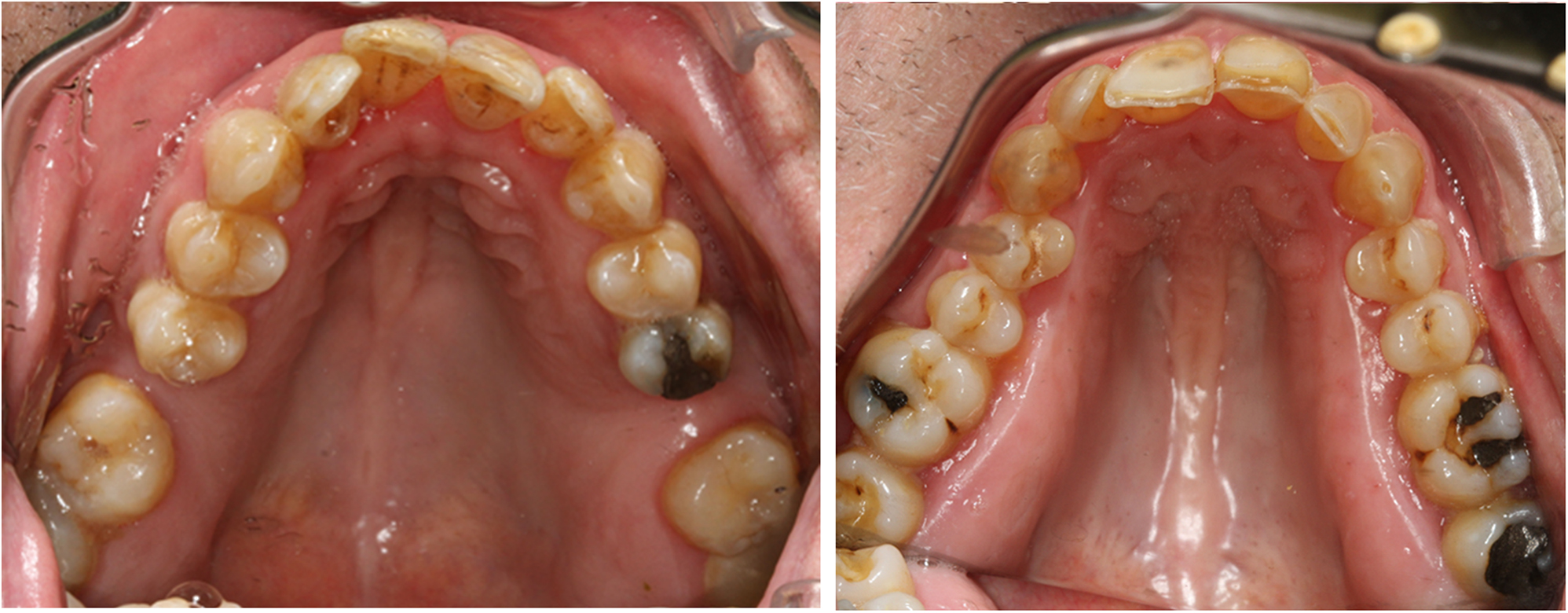
Abnormality of the palate in Bardet-Biedl syndrome. Photographs demonstrating typical high arched palates in BBS

All participants had RP, and their other main features were postaxial polydactyly (26 of 28, 93%), central obesity (23 of 28, 82%), learning difficulties (21 of 30, 70%) and hypogonadism (11 of 30, 37%). Seven of 15 males (47%) reported having a history of hypogonadism and/or genitourinary abnormalities (testicular retention, penile curvature, hypogonadism). Four of 15 females (27%) reported having genitourinary abnormalities (vaginal atresia and urinary tract abnormalities). Oral/dental abnormalities were observed in 20 of 26 participants (77%). Brachydactyly was identified in 23 individuals (77%), and 14 (47%) had syndactyly. Six (20%) participants had gastrointestinal manifestations (irritable bowel syndrome, Hirschsprung’s disease, Crohn’s disease, cholecystitis, and fatty liver disease). Six participants had neurological disorders (e.g., epilepsy, stroke, tics, and behavioural abnormalities). One participant had a history of cancer.

The clinical characteristics and blood test results of the BBS sample, including weight, hypertension status, renal disease status, and diabetes mellitus status, are presented in Table 2. Two participants with a BMI above 30 were on weight-controlling medication, and three others had undergone a gastric sleeve operation to reduce their weight. Blood pressure was measured in 26 participants, but it could not be measured in two participants since the arm cuff was too small and blood pressure records were missing for two participants. Thirteen participants had a SBP above 140 mmHg and/or a DBP above 90 mmHg, including six who were on blood pressure-reducing medication. Five participants who were on blood pressure-reducing medication had a blood pressure that was normal (< 140 mmHg SBP, < 90 mmHg DBP).

Six participants were on cholesterol-lowering medication, two of whom had cholesterol above 6 mmol/L. Five participants reported having cardiovascular disease (i.e., a small atrio-septal defect with a left and right shunt and a slightly dilated right ventricle, aortic stenosis, dilated aorta, and atherosclerosis). One of these individuals was reported to have coronary artery involvement.

Eight participants had renal disease/anomalies (five males and three females), which included prenatally described kidney abnormalities, increased kidney-related blood parameters, kidney failure, and kidney tumour in need of regular follow-up. Three participants with end-stage renal disease were on dialysis, two of whom were awaiting transplantation. One individual had undergone kidney transplantation as a child. These four participants had increased creatinine levels over the sex-based reference (i.e., 105 µmol/L for males and 90 µmol/L for females), increased levels of parathyroid hormone (PTH), increased levels of carbamide, a reduced estimated glomerular filtration rate (eGFR) below 60 ml/min/1.73 m^2^, and normal calcium levels. Two other individuals had increased creatinine levels and reduced eGFRs. One of these two individuals had regular follow-ups of kidney function while the other was unaware of reduced kidney function.

Seven participants (23%) reported having been diagnosed with diabetes, of whom five used medications for diabetes. Five participants had an HbA1c > 42 µmol/mol and reported having diabetes, two of whom had an HbA1c > 53 µmol/mol.

Most participants had normal thyroid function. Two females and one male had TSH levels above 4.0 × 10E-3 IU/L, while their free T4 levels were normal. One individual had a low TSH of 0.05 × 10E-3 IU/L and an increased free T4 of 27 pmol/L but had not been diagnosed with hyperthyroidism. One individual had increased free T4 but normal TSH and free T3. One participant reported having hypothyroidism and was on thyroid medication. Other endocrine abnormalities included polycystic ovary syndrome (*n* = 1) and growth hormone deficiency (*n* = 1).

One participant with a diagnosed fatty liver had increased alanine aminotransferase (ALAT) above 45 U/L, increased aspartate aminotransferase (ASAT) above 70 U/L, and increased lactate dehydrogenase (LD) above 205 U/L. Another participant had also been diagnosed with a fatty liver; in this individual LD was not increased, but ASAT and ALAT measurements were not available.

Five individuals (16%) reported having both anxiety and depression, four individuals reported having anxiety, and one had a history of depression during the last 12 months.

**Table 2:** Clinical characteristics, medical information and laboratory findings

Variables	Total sample (*n* = 30)
**Hypertension**^1^, **BMI**^2^**and body fat parameters**	
Normal weight (BMI > 18,5 but < 25 kg/m^2^)	2 (7)
Overweight (BMI ≥ 25 kg/m^2^ but < 30 kg/m^2^)	5 (17)
Obesity (BMI ≥ 30)	23 (76)
Blood pressure medication	13 (43)
Hypertension	20 (67)
SBP (mmHg) (*n* = 26)	134.1 ± 20.2
DBP (mmHg) (*n* = 26)	85.5 ± 14.26
Total cholesterol, ↑ 5 µmol/L (*n* = 28)	15 (54)
LDL, ↑ 3 µmol/L (*n* = 27)	27 (90)
LDL, ↑ 5 µmol/L (*n* = 27)	16 (59)
Raised triglycerides, ↑ 1.7 µmol/L (*n* = 27)	14 (52)
Raised triglycerides, ↑ 2.6 µmol/L (*n* = 27)	6 (22)
**Kidney disease**	**8 (27)**
Dialysis prior to study	3 (10)
Creatinine, ↑ range ^3^ (*n* = 27)	8 (30)
Creatinine (µmol/L) in those with kidney disease (*n* = 6) ^4^	426.3 ± 333.4
Creatinin (µmol/L) in those without known kidney disease	80.8 ± 13.7
eGFR, ↓ 60mL/min/1.73m2 (*n* = 27)	6 (22)
eGFR (mL/min/1.73 m2) in those with kidney disease	33.5 ± 37.0
eGFR (mL/min/1.73 m2) in those without kidney disease	89.8 ± 19.6
**Type 2 diabetes mellitus** ^5^	**7 (23)**
HbA1c, ↑ 42 µmol /mol (*n* = 28)	5 (18)
**Psychiatric history in the past 12 months**	10 (33)
Anti-depressive medication	4 (13)

Data are presented as *n* (%) or mean ± standard deviation

Abbreviations. BMI = Body mass index; LDL = Low-density lipoprotein; eGFR = Estimated glomerular filtration rate; HbA1c = glycated haemoglobin

^1^ Hypertension defined as systolic blood pressure (SBP) above 140 mmHG and/or diastolic blood pressure (DBP) above 90 mmHG or being on antihypertensive medication

^2^ BMI = weight in kilograms divided by the square of height in meters

^3^ Creatinine above 105 micromol/L for men and above 90 micromol/L for woman

^4^ Two participants with kidney disease did not deliver blood samples

^5^ Insulin, glucose and C-peptide were measured but data are not included because several of the samples had become too old for the analysis and/or individuals had not fasted

“↑”means above the given value, “↓”means below the given value

Visual acuity was analysed in 26 participants (see Table 3), and ERG was performed in 11 participants. Extinguished ERG was present in all 11 individuals. Fourteen participants had previously had an extinguished ERG, and one participant was unable to attend the ERG appointment. Five individuals had undergone surgery for cataract and had intraocular lenses, four of these individuals had bilateral secondary cataract and another fifteen participants had posterior cataract. Macular atrophy and retinal pigment epithelial changes were observed in all participants (*n* = 26), corresponding with their condition of RP.

**Table 3:** Ophthalmic features observed in 26 adults with Bardet-Biedl syndrome

	Total sample (*n* = 26) *n* (%)	
Bilateral secondary cataract present	4 (15)	
Bilateral posterior polar cataract present	15 (58)	
Clear lens	6 (23)	
Intraocular lenses	5 (19)	
Nystagmus present	6 (23)	
Squint	16 (62)	
Macular degeneration present	26 (100)	
Retinal pigment epithelial changes	26 (100)	
Optic disc pallor	25 (96)	
**Visual acuity Best-corrected visual acuity**	**Right eye**	**Left eye**
Finger counting 0.5–2.0 m	3 (12)	5 (19)
Hand motion	4 (15)	6 (23)
Perception of light	13 (50)	13 (50)
No perception of light	1 (4)	1 (4)
Vision 0.05–0.5 m	5 (19)	1 (4)

A straight facial profile was observed in the majority of the participants (see Table 4). An abnormal palate was observed in 58% of the participants (38% had a high palate and 18% had a high and narrow palate). Open bite was seen in 16% (both frontal and lateral 8%) and crossbite in 16% (unilateral 12%, bilateral 4%). Crowded teeth were observed in half of the sample; six were classified as mildly crowded teeth (< 2 mm), six as moderate (2–4 mm), and one as severely crowded teeth (> 4 mm).

**Table 4:** Extra- and intraoral manifestations of 26 adults with Bardet-Biedl syndrome

	Total sample (*n* = 26) *n* (%)
Straight facial profile (normal)	22 (85)
Convex facial profile	4 (15)
Concave	0
Increased mandibular angle	3 (12)
Abnormal palate morphology	15 (58)
Overbite (≥ 4 mm)	11 (44)#
Overjet normal (2–4 mm)	12 (48)#
Overjet increased (> 4 mm)	8 (32)#
Overjet decreased (< 2 mm)	5 (20)#
Open bite	4 (16)#
Cross bite	4 (16)#
Crowded teeth (mild to severe)	13 (50)
Excessive space in the tooth arch	4 (16)#
Small teeth/short roots	15 (60)#

Note. # *n* = 25

### Genetic results

Twenty-eight (28/30) participants provided blood samples for genetic analysis. One had a confirmative genetic test analysed within the last five years and this case is included when discussing the analysed samples and it is listed in Table 5. The other individual that did not provide a blood sample had siblings with BBS who participated in this study, and they were found to be homozygous for c.1169T > G p.(Met390Arg) in *BBS1;* this individual is not included in Table 5. The results of previous genetic testing were available for 17 participants. Some variants known to be related to BBS had been detected; however, these variants had not been evaluated according to the American College of Medical Genetics criteria [31]. When comparing the present genetic analyses results with the previous genetic results (*n* = 17), we identified identical gene variants in 11 participants (*BBS10 n* = 6; *BBS1 n* = 5) and novel gene variants in six participants. Nine participants (31%) had not been genetically tested previously.

A genetic cause or probable cause was identified in 29 participants (see Table 5). Most participants had pathogenic or likely pathogenic variants in *BBS1* (*n* = 11) or *BBS10* (*n* = 9). Homozygosity for c.1169T > G p.(Met390Arg) in *BBS1* was identified in eight participants (28%), and homozygosity for c.271dup p.(Cys91Leufs*5) in *BBS10* was identified in eight participants (28%). Four participants had likely pathogenic variants in *BBS7*, two participants in *MKKS*, two in *BBS9*, and one in *BBS5*. In two of three individuals for whom previous genetic testing had not identified the genetic cause, exome sequencing identified only one heterozygous variant in *BBS7*, c.649dup. By genome sequencing and manual examination of the intronic sequence, we identified a deep intronic variant, c.1037 + 522_1037 + 523delinsAA, in *BBS7*. The third participant with negative exome sequencing data was homozygous for this deep intronic variant. An mRNA analysis of this *BBS7* variant in periphery blood cells from the homozygous individual revealed an insertion of a pseudoexon, r.1037 + 310_1037 + 519ins, in the *BBS7* mRNA (see Additional files 1 and 2). The inserted sequence contains a termination codon p.(346Alafs2*) and the mutated mRNA will likely be degraded by nonsense-mediated decay.

**Table 5:** Genetic variants in 29 adults with Bardet-Biedl syndrome

Gene	Transcript	*n* (%)	Nucleotide change	Protein change	Published or novel	Homozygous or assumed or confirmed compound heterozygous	ACMG class ^a^
**BBS1**	**NM_024649.5**	**11 (38%)**					
		8	c.1169T > G	p.Met390Arg	(44)	Homozygous	*P*
		1	c.1110 + 3G > C	p.(?)	(47)	Homozygous	LP
		1 ^b^	c.1169T > G c.1110 + 3G > C	p.Met390Arg p.(?)	(44) (47)	Assumed compound heterozygous	P LP
		1	c.667 A > C c.1169T > G	p.Thr223Pro p.Met390Arg	Novel (44)	Confirmed compound heterozygous	LP P
***BBS5***	**NM_152384.3**	**1 (3%)**					
		1	c.265 C > T	p.Arg89*	(57)	Homozygous	P
***MKKS***	**NM_018848.3**	**2 (7%)**					
		1	c.-417-1G > T c.515_516del	p.(?) p.Glu172fs*19	Novel (52)	Assumed compound heterozygous	P P
		1	c.792T > G c.1272 + 1G > A	p.Tyr264* p.(?)	(49) (50)	Confirmed compound heterozygous	P P
***BBS7***	**NM_176824.3**	**4 (14%)**					
		1	c.1677-1G > A	p.(?)	Novel	Homozygous	P
		1	c.1037 + 522_1037 + 523delinsAA	p.(?)	Novel	Homozygous	LP
		2	c.649dup c.1037 + 522_1037 + 523delinsAA	p.Ala217Glyfs*19 p.(?)	Novel Novel	Confirmed compound heterozygous	P LP
***BBS9***	**NM_198428.3**	**2 (7%)**					
		1	c.598 C > T c.1329 + 3 A > G	p.Gln200* p.(?)	Novel Novel	Assumed compound heterozygous	P LP
		1	c.214del]	p.Val72Trpfs*12	(52)	Homozygous	P
***BBS10***	**NM_024685.4**	**9 (31%)**					
		8	c.271dup	p.Cys91Leufs*5	(56)	Homozygous	P
		1	c.271dup c.1220T > G	p.Cys91Leufs*5 p.Ile407Ser	(56) Novel	Confirmed compound heterozygous	P LP

Abbreviations: ACMG: American College of Medical Genetics (Classification of variants). P = Pathogenic; LP = Likely pathogenic

The gene name, total number of individuals per gene and the transcript used are in bold as well as the headings of each column

^a^ Based on Richards et al. (2015). ACMG Laboratory Quality Assurance Committee. Standards and guidelines for the interpretation of sequence variants: a joint consensus recommendation of the American College of Medical Genetics and Genomics and the Association for Molecular Pathology. Genet Med. 2015 May;17 [5]:405 − 24. doi: 10.1038/gim.2015.30. Epub 2015 Mar 5. PMID: 25,741,868; PMCID: PMC4544753

^b^ Genetic analysis was conducted close to study start and was therefore included

## Discussion

In this multidisciplinary and genetic study, we investigated the clinical, ophthalmological, oral, and genetic characteristics of 30 adults diagnosed with BBS in Norway. Variants in *BBS1* and *BBS10* were found in 69%, consistent with findings reported in other European countries [26, 34]. All participants had developed RP, which is in line with previous reports [19, 25] and extinguished ERG responses were found in all of those analysed. In addition to ophthalmological findings, abnormal palate morphology and crowded teeth were present in approximately half of the BBS individuals, similar to findings reported elsewhere [8, 11]. The presence of postaxial polydactyly (93%), central obesity (82%), and learning difficulties (70%) were significant features, confirming previously published reports [1, 8, 11].

Obesity was a dominant clinical feature, as 76% of the participants were obese (BMI ≥ 30), which is similar to the findings of earlier studies on BBS [8, 23, 32, 33]. Morbid obesity (BMI above 40 kg/m^2^) was found in one-third of the sample, which was higher than that previously reported [12]. In 2022, the antiobesity medication setmelanotide, an injectable melanocortin 4 receptor agonist, was approved by the FDA for individuals with BBS [34]. None of our participants were using setmelanotide, as it has not yet been approved in Norway. Over 95% of children with BBS over six years of age are overweight or obese [7]. The published data on setmelanotide in children and adults with BBS suggest that 52 weeks of treatment could improve quality of life and reduce body weight and hunger [22, 35], although further research is needed to determine whether the effects of weight loss are maintained in the long term.

Obesity in the general population increases the risk of heart disease, hypertension, type 2 diabetes, and several cancers [36]. Other studies have shown that obesity and hypertension are more common in individuals with BBS than in the general population [20, 23]. The prevalence of hypertension was 67% in our BBS sample, similar to the findings of an earlier study in a BBS population [23]. Type 2 diabetes mellitus was present in 23% of the participants in this study, which is comparable to the 16% reported elsewhere in young adults [23], although a higher rate (48%) has been reported in individuals with BBS at a similar age as in our sample [12].

Kidney failure is still one of the major causes of morbidity in BBS and gives rise to the need for transplantation [14]. Our data showed that 27% of the participants had renal disease or anomalies, and 10% had known end-stage renal disease, similar to earlier reports of 8–14% [16, 37, 38]. Kidney disease has been described as more prevalent in *BBS2-*, *BBS7-*, and *BBS9-*related BBS [1, 6], as well as in *BBS6-*,* BBS10-*, and *BBS12-*related BBS [14, 20]. In the present study, end-stage renal disease requiring dialysis was identified in one participant with *BBS7-*related BBS, one with *BBS9*-related BBS, and one with *BBS1*-related BBS. All participants with kidney failure (*n* = 3) and the one who had undergone kidney transplantation in childhood had parathyroid hormone levels, creatinine levels, eGFRs, carbamide levels, and calcium levels that could indicate secondary hyperparathyroidism, which is commonly observed in individuals with kidney failure. Furthermore, one participant had creatinine and eGFR levels classified in the range of mildly to moderately decreased kidney function [39] without being aware of it.

Increased TSH levels were observed in 11% of our sample, which could be caused by subclinical hypothyroidism, as has been previously reported in BBS [23].

Vision deterioration can be the first symptom of BBS, and ophthalmologists play an important role in the early diagnosis of BBS [36]. In this study, all examined participants had severe retinal dystrophy, macular atrophy, and retinal pigmentary epithelial changes. These ophthalmic features are less frequent in younger individuals with BBS [40]. Nystagmus was present in 23% of the participants, similar to earlier reports [40].

Hypodontia, microdontia, a high arched palate, and crowding are common findings in BBS, and oral/dental changes constitute a minor clinical feature [8, 17]. Taurodontism has previously been described in 83% of individuals with BBS [41]. Our participants commonly had dental anomalies such as microdontia, short roots, and crowding and spacing problems, consistent with Penny et al. [17]. The majority of participants exhibited overjet values within the normal range, yet some of them had an increased overbite. Hypodontia is difficult to evaluate in an adult population as it is challenging to determine its underlying cause. Therefore, we did not include hypodontia when we decided whether the oral/dental criterion was present or not, but in a paediatric population, hypodontia might be of greater value as part of the dental/oral criteria.

The clinical diagnosis of BBS relies on the presence of major and minor features [6]. Age at diagnosis showed great variation in our sample, with the oldest being 30 years at diagnosis. With the increasing availability of genetic testing, the age at diagnosis should decline for future generations. With a genetically confirmed diagnosis, future knowledge on genotype‒phenotype correlations might make it easier to tailor the management to each individual. One of the major drivers in the development of personalized medicine is genomic research in clinical medicine and health care [13, 42].

Previous genetic testing did not identify the genetic cause in six of the 17 previously tested individuals. Three of these were analysed more than ten years ago and the tests involved the identification of specific variants in known BBS genes, such as *BBS1* and *BBS10.* In the three analysed more recently without finding a genetic cause, this was due to exome sequencing not being sufficient to identify the causative variant.

Pathogenic or likely pathogenic homozygous or confirmed or assumed compound heterozygous variants were identified in all of the tested participants, with *BBS1* and *BBS10* as the most commonly associated genes, similar to earlier publications [1, 11, 25]. In previous publications, a probable genetic cause of BBS has been detected in approximately 48–80% of cases [3, 6, 43], depending on the number of genes analysed and the method used. With the use of exome sequencing and gene panels for Bardet–Biedl syndrome or retinal dystrophies, pathogenic or likely pathogenic, homozygous or assumed or confirmed compound heterozygous variants were identified in 89% (26/29) of our participants. With the addition of genome sequencing and manual inspection, a likely pathogenic variant was identified in the remaining three participants.

Variants were found in six different genes in the population of this study (*BBS1*,* BBS5*,* MKKS*,* BBS7*,* BBS9*, and *BBS10*). A total of seven participants had variants identified that have not been reported previously. The most common *BBS1* variant in this study was c.1169T > G p.(Met390Arg). This variant has previously been reported in approximately 80% of individuals with BBS caused by *BBS1* variants [44–46]. One individual in our cohort had this variant in assumed compound heterozygosity together with another known *BBS1* variant, c.1110 + 3G > C, a variant previously reported as a founder mutation in the Faroe Islands [47]. In another individual who was confirmed to be compound heterozygous, in addition to c.1169T > G in *BBS1*, the variant c.667 A > C in *BBS1* was identified. This variant is not listed in gnomAD [48] and has not previously been reported in BBS.

Two participants had variants in *MKKS*; one was confirmed to be compound heterozygous for two variants that previously have been reported as homozygous, c.792T > G [49] and c.1272 + 1G > A [50]. This individual had undergone surgery for vaginal atresia, and the differential diagnosis of McKusick–Kaufman syndrome [51] could be considered, but due to RP, this was ruled out. The other individual with variants in *MKKS* was assumed to be compound heterozygous for one previously undescribed variant, c.-417-1G > T, which affects the splicing of exon 3 in *MKKS* mRNA. Since this is the first coding exon, this will most likely give no functional mRNA from this allele. We have not been able to analyse mRNA from this individual. The other variant, c.515_516del p.(Glu172fs*19), induces a frameshift at position 172 and a premature stop codon 18 downstream. We suspect that this will lead to the degradation of *MKKS* mRNA by nonsense-mediated decay. This variant is registered as pathogenic and likely pathogenic in ClinVar (accession: VCV001457941.7, accessed March 11, 2024) [52], is registered with BBS as the condition, and has no frequency in gnomAD.

The three participants analysed by genome sequencing all harboured the same novel intronic variant in *BBS7*, c.1037 + 522_1037 + 523delinsAA. Two of the participants were confirmed to be compound heterozygous for this deep intronic variant and another previously unreported variant, c.649dup, in *BBS7* and one individual was homozygous for the deep intronic variant. The *BBS7* deep intronic variant c.1037 + 522_1037 + 523delinsAA caused a moderate predicted strengthening of a cryptic donor splice site located at c.-1037 + 519 (Additional file 1). mRNA analysis revealed an insertion of a pseudoexon using the strengthened donor spilce site and, a cryptic acceptor splice site localized at c.1037 + 310. The mRNA analysis is not quantitative and the cDNA sequencing also show correct spliced mRNA, indicating that the mis-splicing is not complete. However, the PCR has a strong bias towards shorter products and the amount of correct spliced mRNA might be highly overestimated. The added sequence contains a stop codon, and the mis-spliced mRNA is therefore likely to be degraded by nonsense-mediated decay.

This deep intronic variant was first identified by manual inspection of the *BBS7* gene from genome sequencing data. This finding supports what has been demonstrated by Varela et al. [53]: that searching for noncoding variants in a targeted manner is an efficient way to identify “missing” variants. The three participants are not known to be related, but all originate from a relatively small region in Norway; therefore, we cannot rule out that they are distantly related.

Two participants had variants in *BBS9*: one was assumed to be compound heterozygous for two previously undescribed variants, c.598 C > T, which changes glutamine at position 200 to a stop codon and probably gives rise to nonsense-mediated decay, and c.1329 + 3 A > G, which is predicted to lead to loss of the donor splice site in exon 12 in *BBS9* mRNA. Neither of these have been reported before and are not listed in gnomAD. The other individual was homozygous for a frameshift variant in *BBS9*: c.214del p.(Val72Trpsfs*12), a variant that has been reported to be pathogenic in ClinVar (accession: VCV000975060.6, accessed March 13, 2024) [52] and has no frequency in gnomAD. It will probably lead to nonsense-mediated decay, as reported for other variants causing the insertion of a premature stop codon in *BBS9* [54].

*BBS10* homozygosity for c.271 dup p.(Cys91Leufs*5) (NM_024685.4) was the most common finding (28%), and this is the most common pathogenic variant reported in *BBS10* in Europe [1, 11, 55]. One individual was confirmed to be compound heterozygous for this variant and for c.1220T > G in *BBS10*; this latter variant, involving the exchange of isoleucine at position 407 with serine, has not been described before as a cause of BBS but has a low carrier frequency in gnomAD (1/21646 Finnish alleles) [48]. Another variant, which changes codon 407 (p.(Ile407Thr)), has been reported to cause BBS [56]. Due to the fulfilment of the clinical criteria in this participant and the finding of these two variants where it was possible to confirm the presence of only one variant in the parental sample analysed, c.1220T > G in *BBS10* was interpreted as likely pathogenic.

Significant adiposity has been described in *BBS10-*related BBS [6]; six of our ten participants with morbid obesity had *BBS10*-related BBS, while three had *BBS1*-related BBS. Unfortunately, our study population was not large enough to evaluate genotype–phenotype conclusions on this topic.

Our study has several limitations. Due to the small sample size, genotype‒phenotype correlations could not be analysed, and because the participants were adults, parents were often not available for phase determination. Unfortunately, waist measurements were not taken to evaluate central obesity, and therefore, we cannot conclude whether the WHO definition is fulfilled. Information on health issues and clinical history was self-reported, but it was verified in medical records. However, it was not possible to obtain medical records for all participants; hence, some information remains unknown, for instance, whether or not polydactyly was present in two individuals. The strengths are that it was possible to identify a probable genetic cause in all analysed participants, the BBS sample was representative of the Norwegian adult BBS population, and we could offer home visits for those who were unavailable to travel.

## Conclusions

In conclusion, multidisciplinary examination of BBS is important for proper management and better outcomes in individuals with this rare genetic disorder. The genotypes and phenotypes of our cohort were heterogeneous and included hypertension, kidney failure, genital anomalies, obesity, and obesity-related phenotypes, specifically diabetes type 2. Physicians should be aware of the clinical complexity of BBS, which necessitates multidisciplinary assessments involving ophthalmologists, nephrologists, geneticists, and many other professionals, such as nutritionists, physical therapists, and psychologists. Although the oral and dental anomalies do not necessarily demand treatment, they may be important findings in the clinical diagnostic process. Other factors, such as increased appetite and reduced vision, may indirectly affect oral health and hygiene. Therefore, a dental team can be important for the follow-up of individuals with BBS. The accumulating genetic data expand our knowledge of the genetic causes of BBS and we highlight the importance of offering genetic testing to individuals who have not been tested and re-evaluating previous genetic testing, examining further for intronic variants when only one variant is found. Genome sequencing can increase the likelihood of identifying the genetic cause of BBS.

## Electronic supplementary material

Below is the link to the electronic supplementary material.

Additional file 1. (a) In silico prediction of the effect of the c.1037 + 522_1037 + 523delinsAA on a cryptic splice site in intron 10 in *BBS7* generated by AlaMut Visual Software version 1.6.1 (SOPHiA GENETICS) including SpliceSiteFinder-like, MaxEntScan, NNSPLICE, GeneSplicer, and Human Splicing Finder. (b) A cryptic acceptor splice site in position c.1037 + 310 and the strengthened donor splice site in position c.1037 + 519 create a possible pseudoexon of 209 nucleotides

Additional file 2. cDNA sequence of *BBS7* amplified from peripheral blood cells of the homozygous individual with the variant c.1037 + 522_1037 + 523delinsAA. The RT-PCR was performed using a forward primer in exon 9 (5’-TCTATCCAGGGTGGTTGTGTAGGA-3’) and a reverse primer in exon 12 (5’-GCTGCTAAAGCTAACAACAGCAGA-3’). The cDNA sequencing was performed using the forward primer in a) and the reverse primer in b). c) A schematical illustration of location of the inserted pseudoexon. d) cDNA sequence of *BBS7* in a control sample

## Data Availability

Norwegian ethical and legal restrictions prevent us from uploading data to public repositories. Norwegian participants with Bardet–Biedl syndrome belong to a relatively small group, and very little personal data are needed to indirectly identify individual study participants. Access to a limited version of the dataset containing selected variables may be made available upon request.

## Abbreviations

ALAT: Alanine aminotransferase
ASAT: Aspartate aminotransferase
BBS: Bardet–Biedl syndrome
BMI: Body mass index
DBP: Diastolic blood pressure
eGFR: Estimated glomerular filtration rate
ERG: Electroretinography
FDA: Food and Drug Administration (USA)
HbA1c: Glycated haemoglobin
LD: Lactate dehydrogenase
LDL: Low-density lipoprotein
PTH: Parathyroid hormone
RP: Retinitis pigmentosa
SBP: Systolic blood pressure
SD: Standard deviation
