# Supplementary material for: Clinical and genetic aspects of Bardet–Biedl syndrome in adults in Norway
Source: Orphanet J Rare Dis. 2025 Mar 14;20:127. doi: 10.1186/s13023-025-03641-3 (PMC11909833; doi:10.1186/s13023-025-03641-3)
Supplement: Supplementary file 1 — Additional file 1. (a) In silico prediction of the effect of the c.1037 + 522_1037 + 523delinsAA on a cryptic splice site in intron 10 in BBS7 generated by AlaMut Visual Software version 1.6.1 (SOPHiA GENETICS) including SpliceSiteFinder-like, MaxEntScan, NNSPLICE, GeneSplicer, and Human Splicing Finder. (b) A cryptic acceptor splice site in position c.1037 + 310 and the strengthened donor splice site in position c.1037 + 519 create a possible pseudoexon of 209 nucleotides [file 13023_2025_3641_MOESM1_ESM.pptx]

## Slide 1
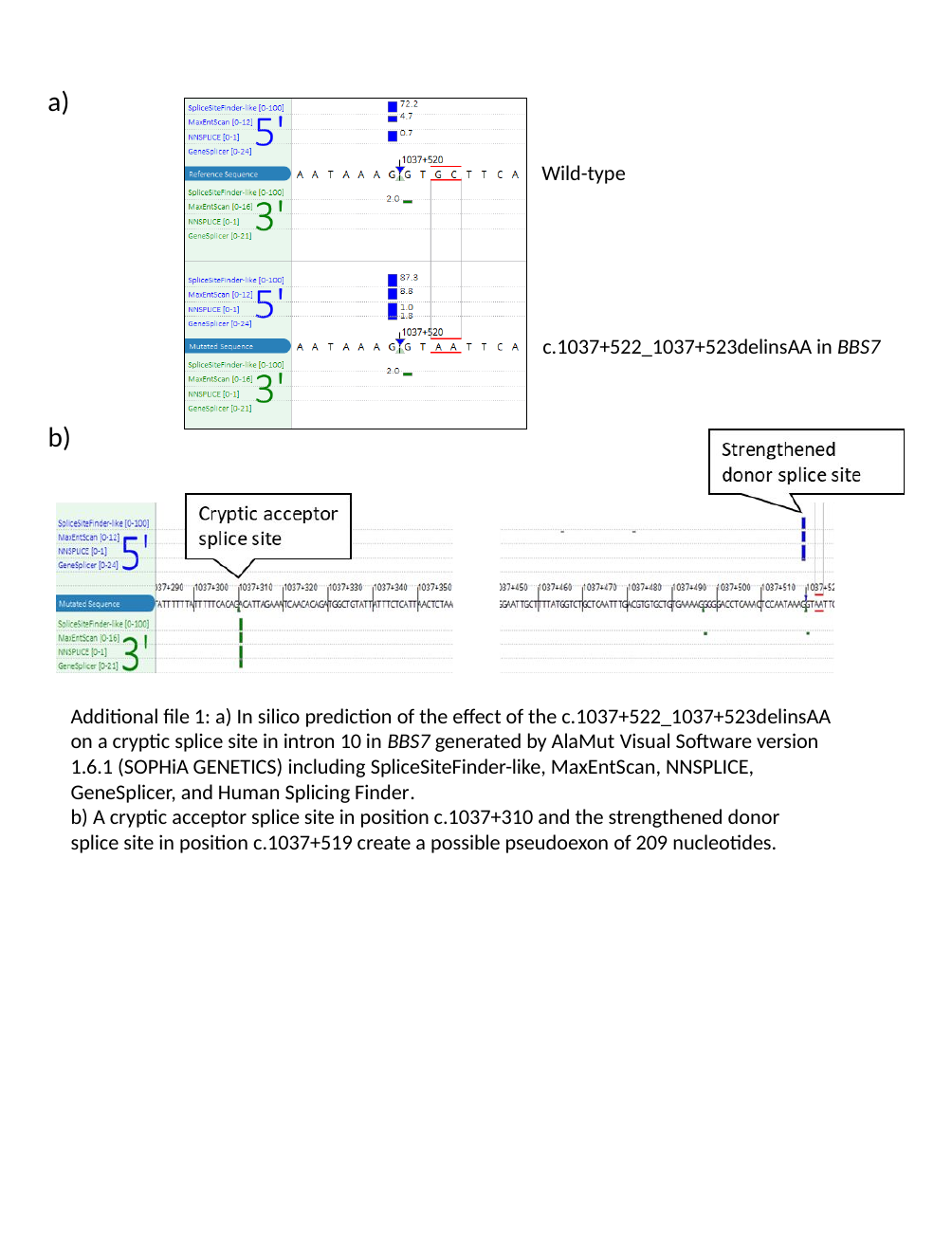

a)
Wild-type
c.1037+522_1037+523delinsAA in BBS7
b)
Additional file 1: a) In silico prediction of the effect of the c.1037+522_1037+523delinsAA on a cryptic splice site in intron 10 in BBS7 generated by AlaMut Visual Software version 1.6.1 (SOPHiA GENETICS) including SpliceSiteFinder-like, MaxEntScan, NNSPLICE, GeneSplicer, and Human Splicing Finder.
b) A cryptic acceptor splice site in position c.1037+310 and the strengthened donor splice site in position c.1037+519 create a possible pseudoexon of 209 nucleotides.
