# Supplementary material for: Clinical and genetic aspects of Bardet–Biedl syndrome in adults in Norway
Source: Orphanet J Rare Dis. 2025 Mar 14;20:127. doi: 10.1186/s13023-025-03641-3 (PMC11909833; doi:10.1186/s13023-025-03641-3)
Supplement: Supplementary file 2 — Additional file 2. cDNA sequence of BBS7 amplified from peripheral blood cells of the homozygous individual with the variant c.1037 + 522_1037 + 523delinsAA. The RT-PCR was performed using a forward primer in exon 9 (5’-TCTATCCAGGGTGGTTGTGTAGGA-3’) and a reverse primer in exon 12 (5’-GCTGCTAAAGCTAACAACAGCAGA-3’). The cDNA sequencing was performed using the forward primer in a) and the reverse primer in b). c) A schematical illustration of location of the inserted pseudoexon. d) cDNA sequence of BBS7 in a control sample [file 13023_2025_3641_MOESM2_ESM.pptx]

## Slide 1
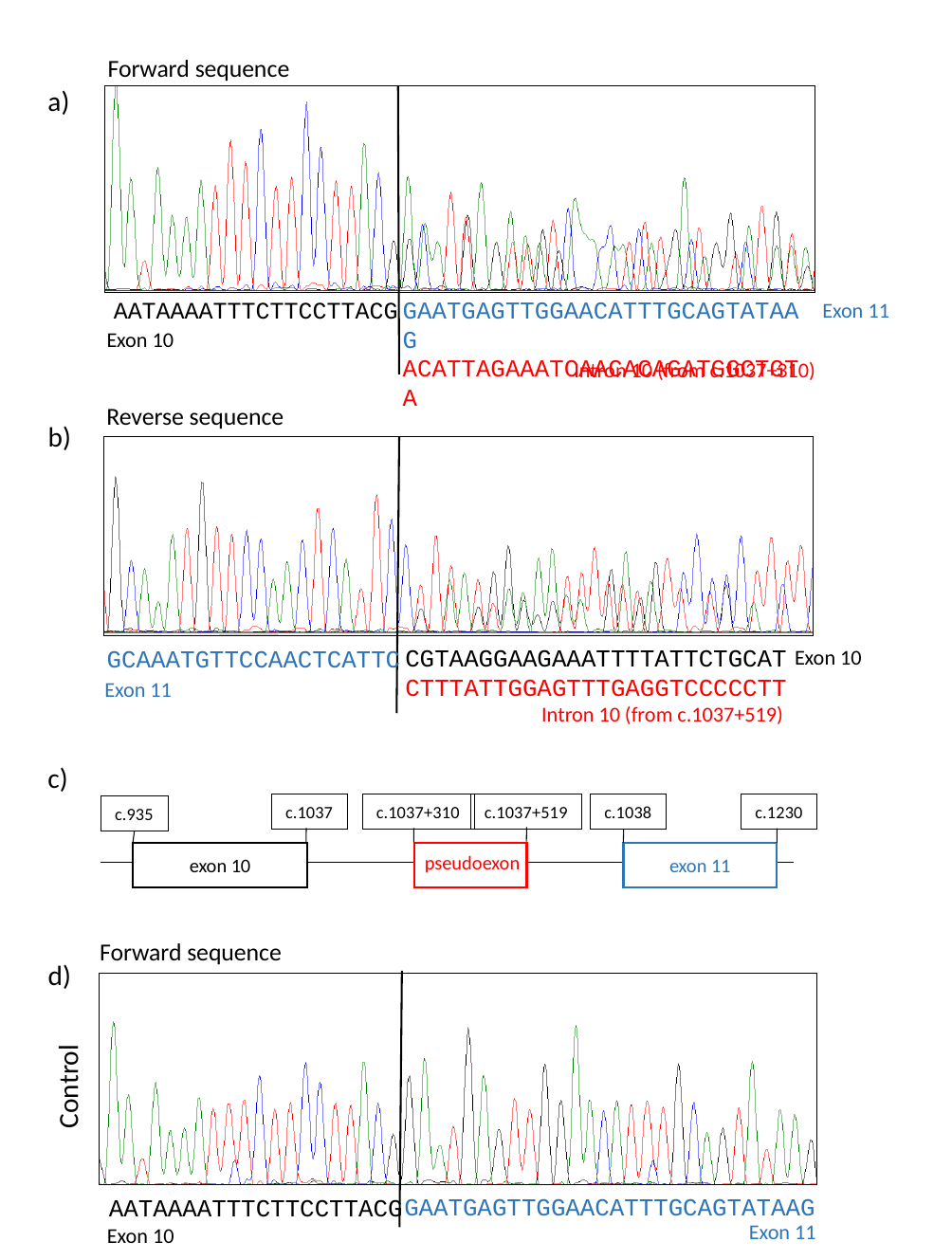

Forward sequence
a)
GAATGAGTTGGAACATTTGCAGTATAAG
ACATTAGAAATCAACACAGATGGCTGTA
AATAAAATTTCTTCCTTACG
Exon 11
Exon 10
Intron 10 (from c.1037+310)
Reverse sequence
b)
CGTAAGGAAGAAATTTTATTCTGCAT
CTTTATTGGAGTTTGAGGTCCCCCTT
GCAAATGTTCCAACTCATTC
Exon 10
Exon 11
Intron 10 (from c.1037+519)
c)
c.1037
c.1037+310
c.1037+519
c.1038
c.1230
c.935
exon 10
exon 11
pseudoexon
Forward sequence
d)
Control
GAATGAGTTGGAACATTTGCAGTATAAG
AATAAAATTTCTTCCTTACG
Exon 11
Exon 10

## Slide 2
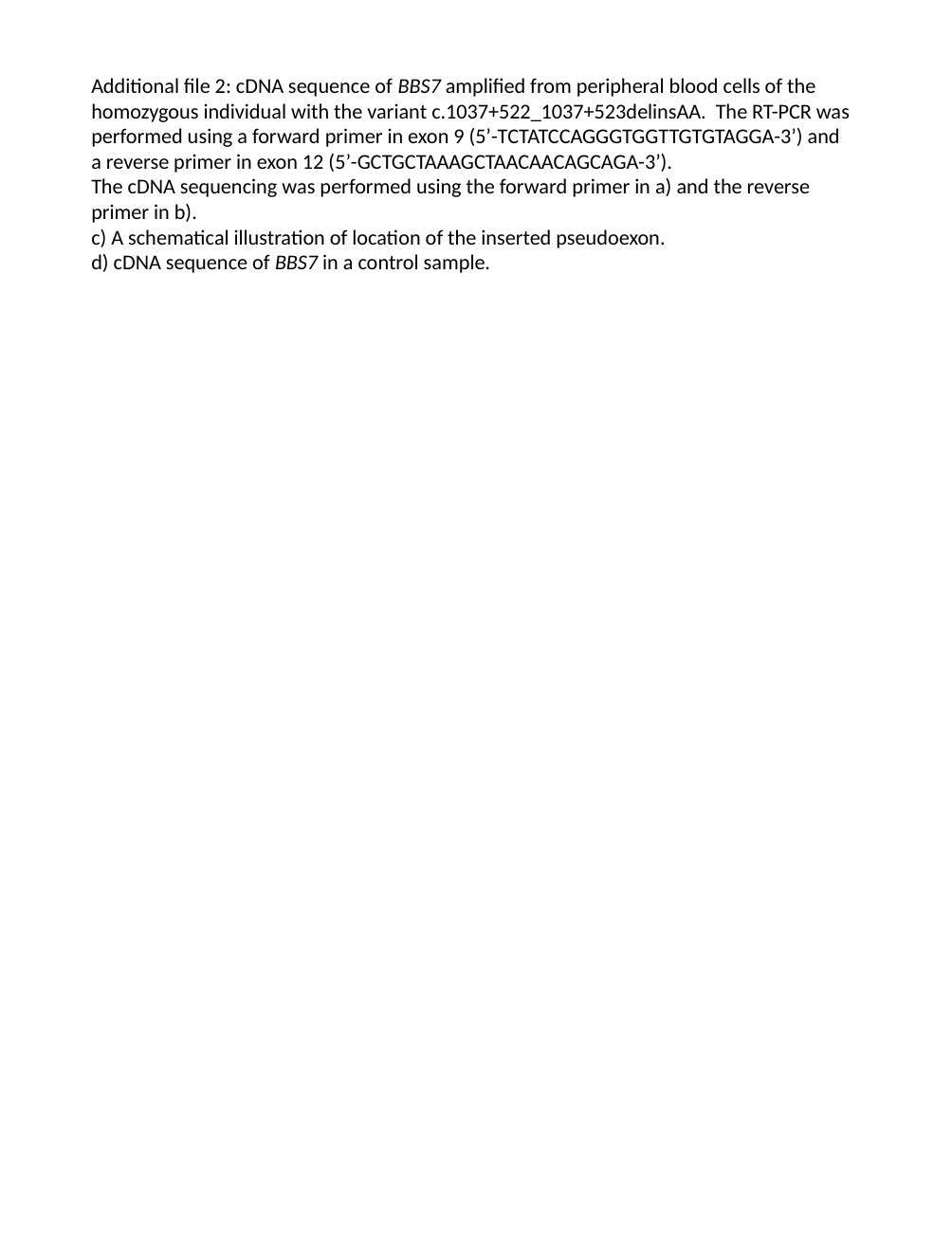

Additional file 2: cDNA sequence of BBS7 amplified from peripheral blood cells of the homozygous individual with the variant c.1037+522_1037+523delinsAA. The RT-PCR was performed using a forward primer in exon 9 (5’-TCTATCCAGGGTGGTTGTGTAGGA-3’) and a reverse primer in exon 12 (5’-GCTGCTAAAGCTAACAACAGCAGA-3’).
The cDNA sequencing was performed using the forward primer in a) and the reverse primer in b).
c) A schematical illustration of location of the inserted pseudoexon.
d) cDNA sequence of BBS7 in a control sample.
